# Analyzing patients satisfaction level for medical services using twitter data

**DOI:** 10.7717/peerj-cs.1697

**Published:** 2024-01-09

**Authors:** Muhammad Usman, Muhammad Mujahid, Furqan Rustam, EmmanuelSoriano Flores, Juan Luis Vidal Mazón, Isabel de la Torre Díez, Imran Ashraf

**Affiliations:** 1 Khwaja Fareed University of Engineering and Information Technology, Rahim Yar Khan, Pakistan; 2 Department of Software Engineering, University of Management & Technology, Lahore, Lahore, Pakistan; 3 Universidad Europea Del Atlantico, Santander, Spain; 4 Department of Project Management, Universidad Internacional Iberoamericana (UNINI-MX), Campeche, Mexico; 5 Universidade Internacional do Cuanza, Municipio do Kuito, Bairro Sede, Angola; 6 Unviersity of Valladolid, Valladolid, Spain; 7 Information and Communication Engineering, Yeungnam University, Gyeongsan si, South Korea

**Keywords:** Health care, Patient satisfaction, Feature selection, Machine learning

## Abstract

Public concern regarding health systems has experienced a rapid surge during the last two years due to the COVID-19 outbreak. Accordingly, medical professionals and health-related institutions reach out to patients and seek feedback to analyze, monitor, and uplift medical services. Such views and perceptions are often shared on social media platforms like Facebook, Instagram, Twitter, *etc*. Twitter is the most popular and commonly used by the researcher as an online platform for instant access to real-time news, opinions, and discussion. Its trending hashtags (#) and viral content make it an ideal hub for monitoring public opinion on a variety of topics. The tweets are extracted using three hashtags #healthcare, #healthcare services, and #medical facilities. Also, location and tweet sentiment analysis are considered in this study. Several recent studies deployed Twitter datasets using ML and DL models, but the results show lower accuracy. In addition, the studies did not perform extensive comparative analysis and lack validation. This study addresses two research questions: first, what are the sentiments of people toward medical services worldwide? and second, how effective are the machine learning and deep learning approaches for the classification of sentiment on healthcare tweets? Experiments are performed using several well-known machine learning models including support vector machine, logistic regression, Gaussian naive Bayes, extra tree classifier, k nearest neighbor, random forest, decision tree, and AdaBoost. In addition, this study proposes a transfer learning-based LSTM-ETC model that effectively predicts the customer’s satisfaction level from the healthcare dataset. Results indicate that despite the best performance by the ETC model with an 0.88 accuracy score, the proposed model outperforms with a 0.95 accuracy score. Predominantly, the people are happy about the provided medical services as the ratio of the positive sentiments is substantially higher than the negative sentiments. The sentiments, either positive or negative, play a crucial role in making important decisions through customer feedback and enhancing quality.

## Introduction

The healthcare industry experienced a rapid surge due to the recent outbreak of the COVID-19 pandemic. Consequently, both the medical institutions and the public have more concerns regarding the quality of medical services. The healthcare industry is focusing on enhancing patients’ experiences by offering high-quality services. The medical services are monitored, analyzed and feedback is requested from the patients. Such feedback is often shared *via* social media platforms like Facebook, Twitter, Instagram, *etc*. The feedback contains users’ views, opinions, and perceptions regarding specific services often accompanied by suggestions and criticism. The views and opinions of users can be analyzed for user sentiments and utilized by medical professionals and institutions alike to uplift the services. Sentiment analysis can play a central role in this regard.

Sentiment analysis has recently been used in the healthcare industry and provides a competitive advantage by better understanding and improving the patient experience (Lai & Mafas, 2020). Many patients utilize the internet to discuss their healthcare service experiences (Yadav et al., 2018). Unprejudiced opinions and reviews from patients are critical for healthcare service providers to improve the quality of services (Hu et al., 2019). Physicians can create interactive patient strategies, such as direct patient involvement, using social media technologies. Doctors may reach out to potential patients and provide information about their medical organization or private practice using social media platforms such as Facebook, Instagram, and Twitter. Among health professionals, 59.3% used one or more social media sites, out of which 43.1% used Facebook, 38.6% used YouTube, 35.9% used LinkedIn, and 22.9% used Twitter. Among these healthcare respondents, 26.8% used social media platforms for health purposes, primarily LinkedIn is used by 70.7% and Twitter by 51.2% (Antheunis, Tates & Nieboer, 2013).

There are many approaches for checking the quality of medical services used in healthcare, especially through sentiment analysis on social media websites like Twitter and patient satisfaction responses. Social media comments and healthcare records could provide crucial information about the quality of health services. Natural language processing (NLP) and machine learning (ML) classifiers are most commonly employed in sentiment analysis to examine the comments posted on social media networks. Sentiment analysis can offer insights into the general public’s views, opinions, and levels of satisfaction by capturing and examining significant feedback, comments, and reviews about medical services (Khanbhai et al., 2021). Esteva et al. (2019) deployed an NLP-based deep learning strategy in healthcare to enhance quality related to health and medicine. This strategy decreases the duration of time a healthcare provider spends on paperwork, providing extra time and the ability to engage with patients immediately during examinations. Because manually evaluating a patient’s medical history or level of satisfaction is laborious and less accurate (Esteva et al., 2019).

However, machine learning-based approaches are very helpful to provide sufficient feedback about any service, and the authorities are able to manage the time and fulfill the medical services. Also, artificial intelligence has brought great innovation to text analysis with various techniques. As compared to machine learning, deep learning can provide accurate results on large numbers of tweets and classify them automatically due to its automatic feature engineering process (Chen & Jain, 2020). Patient satisfaction levels are determined through surveys and questionnaire about their treatments. These surveys produced information about the patients, who were frequently questioned about their experiences with the medical staff, hospital services, and other healthcare-related facilities. Companies in the healthcare industry can learn how to enhance the quality of their services by looking at survey results. The surveys are based on patients’ individual perspectives, which might be influenced by factors such as personal biases, expectations, and unique experiences. The varied rates at which people reply to surveys might potentially contribute to selection bias. Combining surveys with other quality indicators provides the most complete insight into medical care quality (Greaves et al., 2013).

Every country needs to have a comprehensive AI strategy for creating a digital healthcare environment that benefits both health associations and individuals, given the difficulty and expense of developing the various types of artificial intelligence technologies necessary to improve the effectiveness, accessibility, and financial viability of healthcare. The majority of resources currently accessible are focused on enhancing the lives of medical professionals by conducting machine learning research and development on vast amounts of health-related data (Chen & Decary, 2020). Despite the available sentiment analysis techniques, such methods are not generalized and provide different performances with different data sets. Furthermore, the analysis of medical services and patient satisfaction has not been analyzed using patient reviews on Twitter. This study collects a Twitter dataset containing patients’ views and reviews regarding medical services provided by different medical institutions and performs sentiment analysis for investigating the patients’ satisfaction level with those services. Different machine learning models are leveraged for this purpose, accompanied by data preprocessing and feature extraction. Key contributions of the study are:

• A novel dataset related to healthcare service and quality is scraped from Twitter worldwide using the Twitter Tweepy API and is the main feature of our study. To approach different healthcare communities, we utilized three specific keywords, which are “health care”, “health care services”, and “medical facilities”.
• The contributed raw dataset tweets are annotated using two unique lexicon-based techniques, including TextBlob and VADER Lexicon. In addition, we combine these two methods to accurately annotate the dataset and extensively deploy different preprocessing steps to remove redundant information from the tweets.
• The study proposed an LSTM-ETC-based Hybrid method in which we used five layers of the LSTM model to extract features, which were then utilized by the Extra Tree classifier with fine-tuned hyper-parameters to predict the patient’s satisfaction level with superior accuracy.
• This study employed multiple state-of-the-art machine learning approaches for text analysis and sentiment classification in comparison with the proposed approach. To check the reliability and robustness of the proposed approach, we used a cross-validation methodology. Also, we presented the top 15 locations with positive, negative, and neutral sentiments that demonstrate how many patients from specific counties are satisfied or dissatisfied with the services.

The rest of this article is structured as follows: Section  ‘Related Work’ provides a discussion of related studies. Section  ‘Materials and Methods’ presents the study design such as data collection, data preprocessing, sentiment analysis, feature extraction, selection model, and model evaluation. Afterward, the study results are discussed. Finally, the last section concludes our findings of the study.

## Related Work

The wide use of the internet primarily and social media platforms especially has increased the number of e-patients. The number of e-patients is continuously increasing. According to the Pew Research Center’s Internet & American Life Project, between 75% and 80% of Internet users searched for health information online. In addition, 60% say internet information influenced their decision on how to treat a medical problem, 56% say it impacted their overall approach to health management, and 53% say it prompted them to ask a doctor additional questions or seek a second opinion (Susannah, 2008; Lee, 2011). Table 1 presents a summary of systematic analysis studies

**Table 1:** Summary of the systematic analysis studies in related work.

**Study**	**Purpose**	**Model**	**Strengths**	**Limitations**
Ji, Chun & Geller (2013)	Investigate the determinants of positive sentiment expressed in hospital Facebook	NB	SERVQUAL is a tool that is widely used to evaluate the quality of healthcare services, remove barriers, and provide decision-makers clear action consequences.	Just 45 out of 87 hospitals had reviews on Facebook—fake Facebook pages for public hospitals that aren’t authorized or legitimate.
Yang, Lee & Kuo (2016)	Analyzing user-generated contents	LDA	A framework based on LDA to analyze discussion threads in a health community	Sentiment intensities into sentiment analysis have limited opinions for users.
Gopalakrishnan & Ramaswamy, (2017)	Predicting drug satisfaction level	SVM	Create a vector space representation for the drug reviews. The SVM is discovered to have low negative precision.	Only one SVM is used in this study, for better performance need to more models.
Alayba et al. (2017)	Introducing a new Arabic data set on health services	NB	This paper proposes a multi-criteria technique to evaluate and rank Arabic sentiment analysis classifiers empirically. The technique was evaluated using patient reviews in Arabic.	Only positive and negative evaluations were taken into account in this study; neutral, tweets were excluded.
Rahim et al. (2021)	Classifying Facebook reviews of public hospitals	NB	This study is the first to date in Malaysia to develop a machine learning model for evaluating hospital quality of care.	It is focused only on public hospitals not included in private hospitals.
SuryaPrabha & Balakrishnan (2021)	Analyzing the messages on health community posts	SVM	The emotional wordnet technique, which is based on Lexicon, is discussed in this article as a basis for the research of feelings.	The discussion boards of only three disorders affecting women were taken into account. These diseases are not common for every woman.
Saad et al. (2021)	Tracking social perception of healthcare service quality	SVM	A suggested method for tracking and analyzing social views on the caliber of public services.	This study has been limited to one term that was used to gather tweets on medical services.
Htet, Khaing & Myint (2019)	Healthcare related tweets analysis using big data	MaxEnt	Performed improved iterative scaling and a max-entropy classifier with 90% accuracy.	The methodology part and results are not satisfactory.
Izzo & Maloy (2017)	Sentiment analysis for medical students	–	sentiment analysis proved a great variability in the grading system for medical students.	The paper does not utilize performance measures effectively.
Abualigah et al. (2020)	healthcare analysis using sentiments from social media	SVM	They performed analysis on health care related services with high validity.	Only used ML models to perform experiments.
This work	Analyzing the quality of medical services via twitter	LSTM- ETC	This study collects a Twitter dataset containing patients’ medical services. Healthcare quality and views regarding medical services provided by different medical institutions and perform sentiment analysis for investigating the patients’ satisfaction level for those services.	The collected dataset contains the tweets worldwide and it’s a limitation of this study.

Given the benefits it can bring, Twitter is becoming a key component of contemporary medicine, with over 2,000 healthcare practitioners on Twitter who tweet more than once per day and have at least 300 followers. Many tweets from medical accounts provide broad public health information or fresh research on therapies or technologies. Less-experienced physicians, on the other hand, have used Twitter to post remarks about encounters with patients, X-ray images, and cropped photos of real-life patient notes. While certain case studies can be useful to a broad audience, considerable caution must be used to keep identifying information out of them. To avoid privacy breaches, patient data were extracted from tweets. According to one research, more than 5% of tweets from a sample of 500 clinicians with more than 200 followers violated patient privacy (Chretien, Azar & Kind, 2011). These unauthorized accesses to patient information are both unethical and unlawful.

The study by Khan & Khalid (2016) performed aspect-based sentiment analysis on health care data to recommend the best services and treatment by the service providers. The study deploys the machine learning technique, Latent Dirichlet Allocation (LDA) on millions of reviews to perform the analysis. Asghar et al. (2014) proposed an approach for opinion lexicon on medical data extracted from different health forums. The proposed approach is based on the incremental model and tends to show better results than traditional models and achieved 82% accuracy on training data and 78% on testing data. Similarly, Asghar et al. (2013) used a rule-based opinion mining approach for health reviews extracted from health forums. A web crawler is used to extract patient reviews from online healthcare platforms. The approach obtains 89% and 86% accuracy scores on training and testing data, respectively. Similarly, Mujahid et al. (2023) used LDA topic modeling on tweets to extract important topics. Along the same lines, Ramírez-Tinoco et al. (2019) proposed a sentiment analysis approach for health care data. Saad et al. (2021) proposed an approach for sentiment analysis on drug-related reviews using a supervised machine learning approach and lexicon techniques. The deployed approach leverages logistic regression (LR) with a feature union technique by combining term frequency (TF) and term frequency-inverse document frequency (TF-IDF) to achieve a significant 96% accuracy score.

Htet, Khaing & Myint (2019) proposed a big data-based IOT architecture for healthcare-related tweet sentiment analysis using a maximum entropy classifier (MEC). They scrapped data from Twitter using rest API and twitter4J. The collected raw tweets contain meaningless and unnecessary information. Therefore, authors utilized a number of commonly used pre-processing steps including tweets conversion into lowercase, removal of stopwords, tokenization of tweets, convert tweet text into base form. After pre-processing, the authors extract significant features from the tweets with TFIDF feature-engineering approach. TFIDF is commonly employed in a variety of NLP problems. Then used an improved iterative feature vector for normalization. Finally, a max-entropy classifier was used to extract sentiments from the processed tweets. Experiments showed that their proposed method shows good, bad, and fair health status while evaluating the tweets.

Yang, Lee & Kuo, (2016) provided an approach for analyzing data provided by users in various health records. The suggested approach extracts medical terminologies such as diagnoses, symptoms, remedies, and adverse effects from the health records. Following that, the record is organized efficiently based on provided medical statements. The sentiments, feelings, and expressions, towards health status including “physiological and psychological”, are assessed effectively. The suggested approach helps patients to understand the way other patients with comparable problems experience, to get more knowledge about how to give appropriate medical care, and to assess the efficacy of medical treatments (Yang, Lee & Kuo, 2016). A sentiment analysis-based method regarding the health of medical students was developed by Izzo & Maloy (2017). They demonstrate that medical students during pandemic emergencies survive with bounded resources. The textual comments and numerical data were the main concerns in awarding grades. The emotions from the textual comments were extracted through the utilization of an SVM classifier and trained using Twitter comments. The method was 66% accurate for sentiments.

Ji et al. (2015) developed a sentiment analysis-based approach concerning public health in two steps: first, they classified the tweets related to health into two classes (personal and news tweets). Then, identify the negative sentiments in the health tweets that evaluate the level of public health. They differentiate personal tweets from news tweets to enhance the performance of evaluation metrics like recall and precision scores. Moreover, they developed an algorithm that uses p-corpus, which automatically increases the dataset size. They used multiple classifiers to find the best one by performing different experiments on health-related tweets. The naive Bayes (NB) classifier performance was superior for negative tweets on the p-corpus dataset and better than others (Ji, Chun & Geller, 2013). Another article performed healthcare analysis using sentiments from social media to extract and analyze mental knowledge through attitudes and expression. They suggest that sentiment analysis offers many resources and benefits by analyzing medical information to improve healthcare quality (Abualigah et al., 2020).

### Proposed methodology

**Figure 1:**
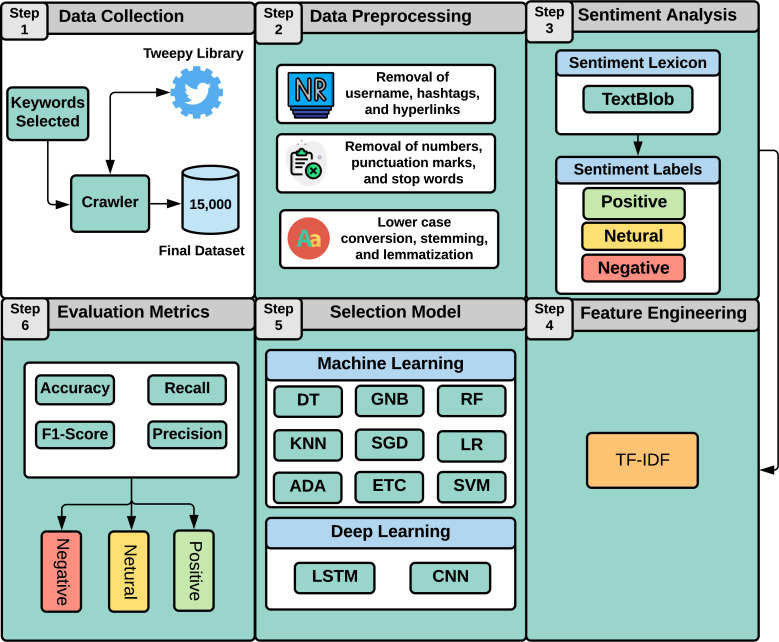
Overview of our study approach.

This study aims at analyzing patients’ satisfaction with the healthcare industry and notifying physicians of the factors causing the lower satisfaction level. It allows physicians to understand what is more important to patients and how well they are interacting with them. Similarly, hospitals may quantify patients’ experiences and find areas for improvement by evaluating the positive and negative comments. As illustrated in Fig. 1, this study employs a machine learning approach to perform sentiment analysis regarding medical services. Our study approach involves six steps as follows:

### Step (1): Data collection

In this step, we discuss the data collection process. We collected our dataset from Twitter using the Tweepy (https://www.tweepy.org/) library. To target different healthcare communities, we utilized three specific keywords, which are “health care”, “health care services”, and “medical facilities” to extract data from Twitter. We extract this data during Covid-19 in July 2021. As the initial dataset, we obtained 5,561 tweets for health care, 6,351 tweets for health care services, and 5,867 tweets for medical facilities. As the tweets for each keyword are not balanced, we randomly selected 5,000 from each keyword. Therefore, the final dataset of our study yielded a total of 15,000 tweets.

### Step (2): Data preprocessing

After collecting the dataset, we need to clean the dataset by performing the complete preprocessing stages to increase the proposed models’ learning performance. The text is converted to lowercase during the preprocessing, followed by stop words and punctuation removal. Next, stemming is performed using the Porter Stemmer (https://tartarus.org/martin/PorterStemmer/) library to obtain the root form of each word. Stemming reduces the complexity of feature space and improves the learning process of the machine learning models (Kotsiantis, Kanellopoulos & Pintelas, 2006; Uysal & Gunal, 2014). Preprocessing steps for text cleaning are

• **Remove number and punctuation:** Number and punctuation are not important to evaluate the sentiment of the text, and they can create unnecessary complexity in the feature set. Thus, we removed them to reduce the complexity of the dataset. We used a regular expression in Python to remove numbers and punctuation.
• **Convert to lowercase:** Dataset can have both upper case and lowercase letters in the text, which increases redundancy in feature sets such as ‘Go’ and ‘go’. These words are both the same, but because of differences in case, they are different for learning models. Thus, converting to lower case technique solves this problem by converting all words later into lower case. We used the tolower() Python function for it.
• **Remove stopwords:** The text contains many meaningless words but makes the sentence more appropriate, such as the, a, an, etc. We remove these words to make the dataset clean without meaningless words. We have done this using python NLTK library (Loper & Bird, 2002).
• **Stemming:** It is also a very worthy technique in which we convert each word into its root form to reduce redundancy in the dataset as go and going are the same meaning words but the difference in the form they are separate features for machine learning model. Stemming will convert ‘going to go’, ‘goes to go’, ‘gone to go’, so complexity can be reduced. We have done this using the Porter stemmer library (Karaa & Gribâa, 2013). Table 2 shows the sample data before and after the processing steps.

### Step (3): Sentiment analysis

**Figure 2:**
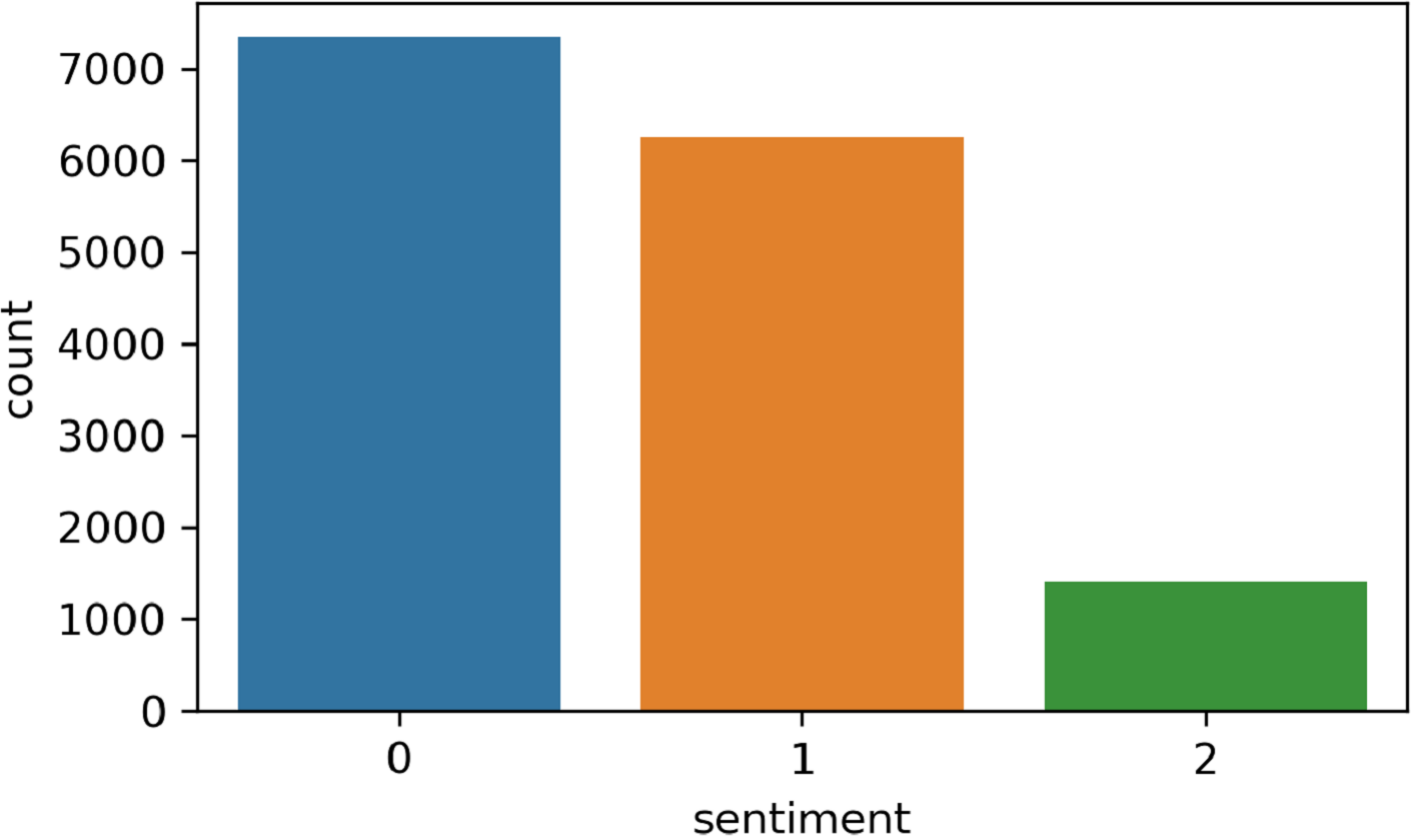
Sentiment count; positive (1), negative (2), and neutral (0).

**Figure 3:**
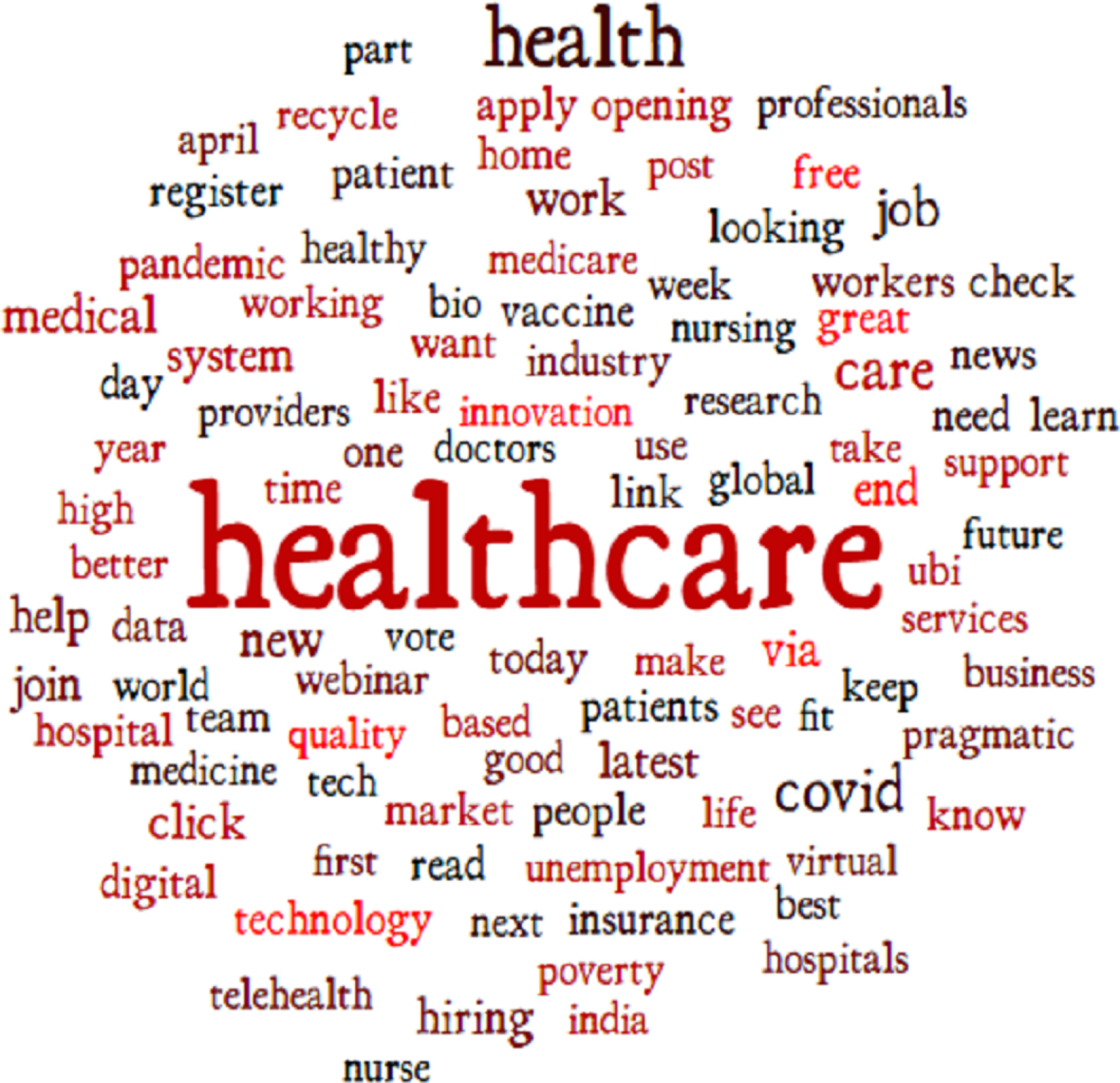
Word cloud of the frequently used words.

In this step, we perform the annotation process to label our dataset. Figure 2 shows the number of samples corresponding to each sentiment in the dataset. The dataset is annotated using the TextBlob (https://textblob.readthedocs.io/en/dev/) library. TextBlob is a popular sentiment analysis lexicon-based Python library model that promises simplified text processing (Sarkar, 2019). The distribution of the most used words in the Tweets dataset is illustrated in Fig. 3 which indicates that ’healthcare’ is one of the most widely used words while giving views on medical services.

**Table 2:** Sample tweets before and after preprocessing.

Original text	Clean text
An exciting opportunity in #healthcare text #analytics, …,	exciting opportunity healthcare text analytics, …,
Modern infrastructure creation will allow Ukraine, …,	modern infrastructure creation allow ukraine, …,
#healthcare #Painrelief #Jointshealth #Proflex, …,	healthcare painrelief jointshealth proflexoral, …,
Join the Covance by Labcorp team! See our latest, …,	join covance labcorp team see latest, …,
We want to help the healthcare ecosystem to become patient, …,	want help healthcare ecosystem become patient, …,

### Step (4): Feature extraction

In this step, we used text data for sentiment classification using a supervised machine learning approach. Machine learning can not directly process the text data as they need numerical representation of text data (Rustam et al., 2020). For that, different feature extraction techniques are available in text mining domains. In this study, we used feature extraction techniques named term frequency-inverse document frequency (TF-IDF).

TF-IDF is used for feature extraction to train the machine learning models. TF-IDF is most widely used in text analysis and music information retrieval (Mujahid et al., 2021). TF-IDF assigns a weight to each term in a document based on its TF and IDF. The terms with higher weight scores are considered to be more important. TF-IDF is a product of TF and IDF, as we explain mathematically below

(1) \begin{eqnarray*}tf=T{F}_{t,j}.\end{eqnarray*}

Here, *tf* is a term frequency of term *t* in document *j*. now IDF can be concluded as:

(2) \begin{eqnarray*}idf=log \left( \frac{N}{{d}_{t}} \right) .\end{eqnarray*}

Here, *N* is the number of documents and *d*_*t*_ is the number of documents containing term *t*. TF-IDF can be defined as

(3) \begin{eqnarray*}tf-idf=T{F}_{t,j}\ast log \left( \frac{N}{{d}_{t}} \right) .\end{eqnarray*}

**Figure 4:**
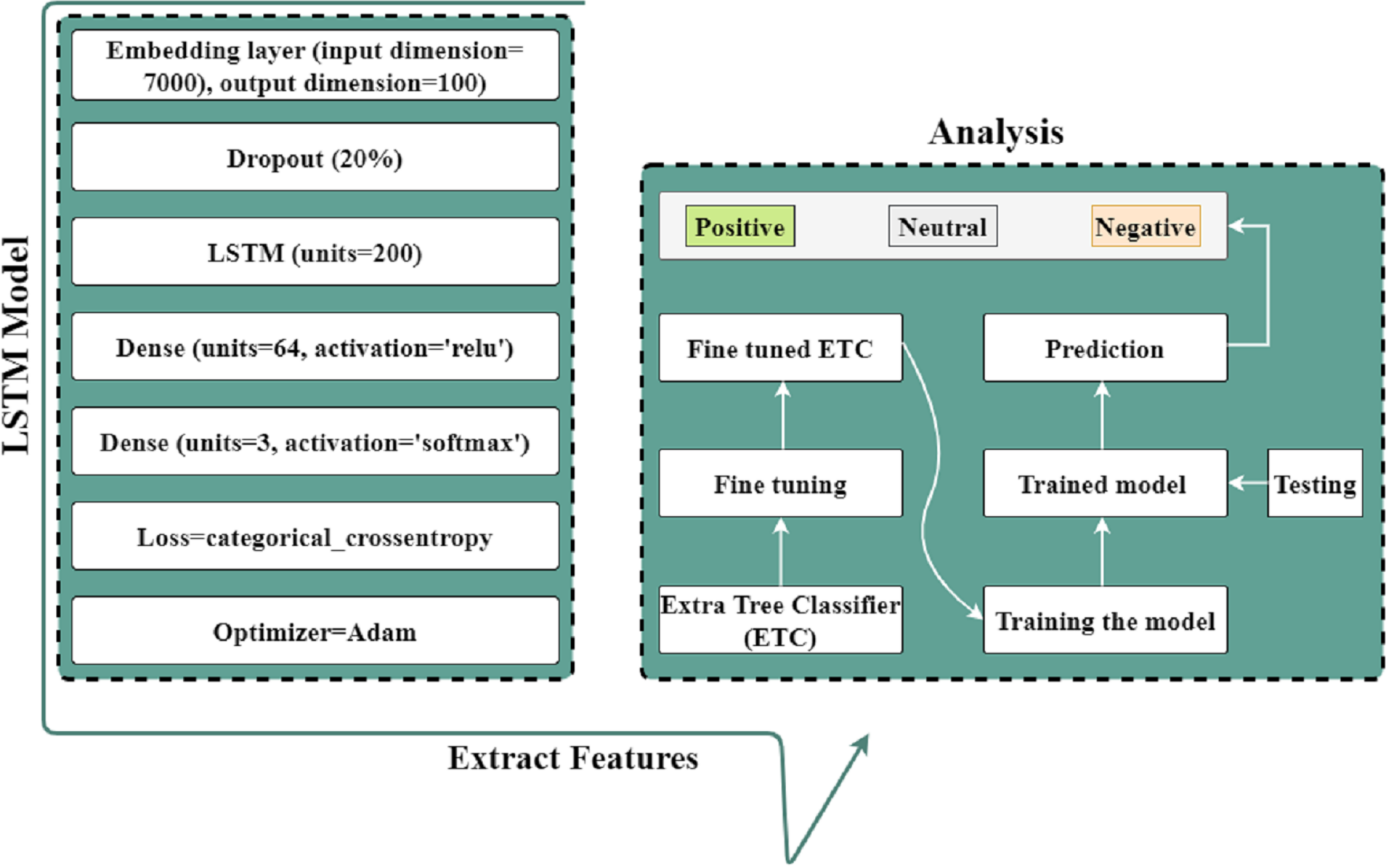
Proposed architecture for analyzing the patient’s satisfaction level.

This study proposed a hybrid approach using the LSTM-ETC model for analyzing patient’s satisfaction level through Twitter data. LSTM is suitable for large datasets to extract valuable features using various layers, whereas ETC is superior for text data. We utilized an embedding layer consisting of 7,000 input dimensions and 100 output dimensions. This embedding layer will receive text data as input and output a numeric representation for LSTM. We employed a five-layer LSTM model, with the second layer containing a 20% dropout rate and 200 LSTM units. The activation function of one dense layer is RELU, while that of the other is SOFTMAX. Then compile and fit the LSTM model with categorical loss to derive valuable features. The extracted features are subsequently utilized by the ETC model to produce extremely accurate predictions. Before making predictions, the ETC model must first be fine-tuned. The proposed architecture for analyzing the patient’s satisfaction level is shown in Fig. 4.

### Step (5): Model selection

This study leverages eight machine learning models for the task at hand. We deployed state-of-the-art models with their best hyper-parameters setting which get using the model tuning. The hyper-parameter setting of these models is shown in Table 3.

**Table 3:** The hyper-parameter settings of machine learning models.

**Models**	**Hyper-Parameters**
RF	n_estimators = 150, max_depth = 150
LR	solver = “saga”, *C* = 2.0
SVM	Kernel = “linear”, *C* = 2.0
DT	max_depth = 150
KNN	n_neighbour = 4
ADA	n_estimator = 150, learning_rate =0.2
GNB	Default setting
SGD	max iter = 200, tol = 1*e*^−3^
ETC	n_estimators = 150, max_depth = 50

#### Decision tree

DT is a training model that can predict the target class based on straightforward rules deduced from earlier data (training data). In DT, for predicting a class name for a record, the process is started from the root (Charbuty & Abdulazeez, 2021). We compare the values of the root property with the record’s quality.

#### Random forest

RF is an ensemble learning method used for classification and regression (Iwendi et al., 2020). It operates by constructing a large number of decision trees during the training and makes the final prediction using the mode of the classes like mean or average prediction (regression) of the individual trees.

(4) \begin{eqnarray*}r{f}_{p}=mode\{ {t}_{1},{t}_{2},{t}_{3},...,{t}_{n}\} .\end{eqnarray*}

#### Extra tree classifier

ETC is a gathering learning strategy on a very basic level based on decision trees (Gupta et al., 2022). ETC follows a similar procedure to RF and randomizes certain choices and subsets of information to reduce over-learning from the information and over-fitting.

(5) \begin{eqnarray*}et{c}_{p}=mode\{ {t}_{1},{t}_{2},{t}_{3},...,{t}_{n}\} .\end{eqnarray*}

#### Logistic regression

Logistic regression (LR) is a statistical analysis method used to predict a data value based on prior observations of the data set (Goswami & Sebastian, 2022). A logistic regression model predicts a dependent data variable by analyzing the relationship between one or more existing independent variables. A logistic function is used by LR for probability approximation. A logistic function, also known as a logistic curve, is a sigmoid or “S” sloped curve that is defined in Eq. (6).

(6) \begin{eqnarray*}SF= \frac{1}{1+{e}^{-({\beta }_{o}+{\beta }_{i})}} .\end{eqnarray*}

#### Gaussian naive Bayes

Gaussian naive bayes (GNB) algorithm is a special type of NB algorithm which is specifically used when the features have continuous values (Ontivero-Ortega et al., 2017). It is also assumed that all the features follow a Gaussian distribution *i.e.,* normal distribution. A Gaussian classifier is a generative approach in the sense that it attempts to model class posterior as well as input class-conditional distribution.

#### Support vector machine

Support vector machine (SVM) is a linear model used for classification and regression tasks (Wu et al., 2020). SVM draws multiple hyper-planes in the features space to classify the dataset. The hyper-plane with the best margin will be used for the classification of data. The SVM classifier’s main goal is to classify data points by estimating the hyperplane using a feature set. The hyperplane’s dimensions change depending on how many features are present. The task is to derive hyperplanes that maximize the margins between samples of classes in n-dimensional space, where hyperplanes have multiple possibilities.

#### K-nearest neighbour

K-nearest neighbour (KNN) is the simplest architecture for a classification model and is easy to implement and interpret (Yigit, 2013). Also known as the lazy learner, it requires fewer computational resources for training and prediction. Often, selecting an appropriate *k* value enhances the performance of KNN where *k* indicates the number of closest neighbors considered to classify a sample. Euclidean distance calculation metric KNN is used to measure the distance given below in Eq. (7):

(7) \begin{eqnarray*}Euclidean~Distance=\sqrt{\sum _{i=1}^{k}({x}_{i}-{y}_{i})^{2}}.\end{eqnarray*}

#### AdaBoost

AdaBoost (ADB) is a tree-based ensemble model for classification and uses decision trees as base model (An & Kim, 2010). ADB ensemble reduces the error during training by joining multiple weak learners to make a strong classifier. ADB assigns a higher weight to the mis-classifier instance and less to already handled well. Boosting is a technique for combining several weak learners into a single composite one. To begin with, it matches the original dataset’s base classifier. Then, on a dataset with more classified instance errors, it trains the other copies of classifiers in a sequential manner.

### Step (6): Model evaluation

We used four evaluation parameters for the machine learning model’s performance measure such as accuracy, precision, recall, and F1 score (Rehan et al., 2021). These evaluation parameters can be calculated using the confusion matrix terms which are true positive (TP), true negative (TN), false positive (FP), and false negative (FN). We define used evaluation parameters below:

**Accuracy**: It is used to measure the correctness of the learning model by dividing the number of correct predictions by the number of total predictions. The accuracy score range is between 0 and 1. Here 0 is the lowest score and 1 is the highest. We can define accuracy as:

(8) \begin{eqnarray*}Accuracy~Score= \frac{total~number~of~correct~predictions}{total~number~of~predictions} .\end{eqnarray*}

or,

(9) \begin{eqnarray*}Accuracy~Score= \frac{TP+TN}{TP+TN+FP+FN} \end{eqnarray*}

here,

• **TP**: When the model prediction example is YES and the actual label of the example is also YES.
• **TN**: When the model prediction example is NO and the actual label of the example is also NO.
• **FP**: When the model prediction example is YES but the actual label of the example is NO.
• **FN**: When the model prediction example is NO the actual label of the example is YES.

**Precision**: It can be calculated as TP divided by the sum of TP and FP. The precision score range is between 0 and 1. Here 0 is the lowest score and 1 is the highest. We can define accuracy as:

(10) \begin{eqnarray*}Precision~Score= \frac{TP}{TP+FP} .\end{eqnarray*}

**Recall**: It can be calculated as TP divided by the sum of TP and FN. The recall score range is between 0 and 1. Here 0 is the lowest score and 1 is the highest. We can define accuracy as:

(11) \begin{eqnarray*}Recall~Score= \frac{TP}{TP+FN} .\end{eqnarray*}

**F1 Score**: It is also known as the F measure. It is a harmonic means of precision and recall. The F1 score range is between 0 and 1. Here 0 is the lowest score and 1 is the highest. We can define accuracy as:

(12) \begin{eqnarray*}F1~Score=2\ast \frac{Precision~Score\ast Recall~Score}{Precision~Score~+~Recall~Score} .\end{eqnarray*}

## Results and Discussions

This section contains the experimental results and analysis for healthcare-related tweets. We deployed lexicon techniques to find the sentiment in the dataset and used machine learning approaches to classify “the tweets as positive negative and neutral”.

### RQ 1: What are the sentiments of people for medical services worldwide?

**Figure 5:**
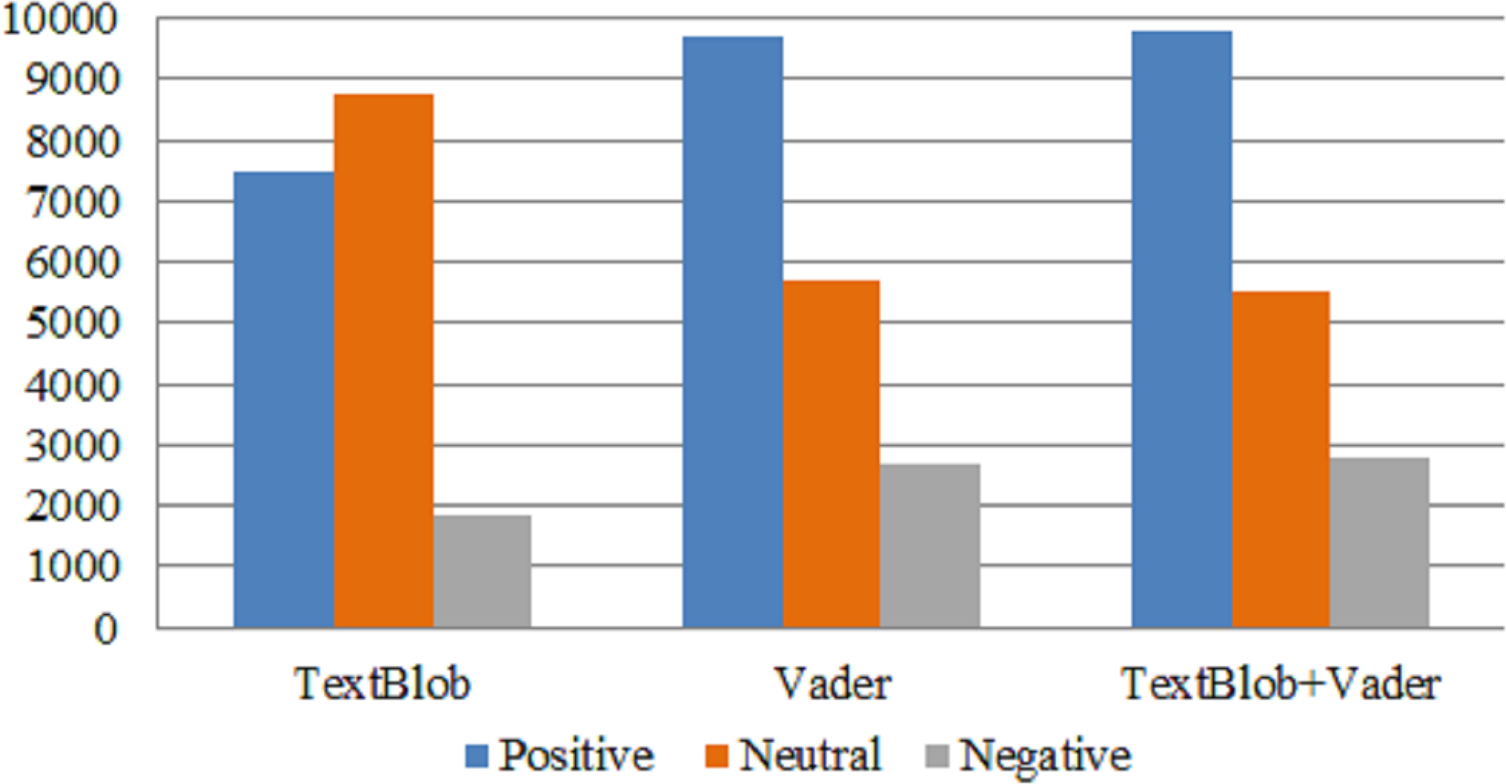
Sentiment count using TextBlob, VADER, and TextBlob+Vader.

This study evaluates the sentiment of people all around the world for the medical facilities provided by the health care departments. We first used lexicon-based techniques to measure the polarity in tweets related to health care. According to the results of TextBlob and Vader ratio of positive sentiment is more as compared to the negative sentiments as shown in Fig. 5. According to the TextBlob results the ratio of positive sentiments in the dataset is 41.62% and negative sentiment 9.4%. While we got some similar results with Vader also in comparison as it find the ratio of positive sentiments 54.44% and negative sentiments 14%. These results show that people are happier towards the facilities in healthcare domains.

TextBlob employs a simple algorithm that is unable to capture complicated emotional nuance. Vader, on the other hand, is designed specifically for social media tweets using a lexicon, grammatical strategies, and pre-trained lexicons. Vader comprehends the negation, intensity, and punctuation of both formal and informal languages. However, we combined TextBlob and Vader to take advantage of their respective strengths and capabilities to accurately label the raw tweets. Figure 5 shows the sentiment count using TextBlob, Vader, and a combination of TextBlob and Vader techniques. The annotated dataset with TextBlob-Vader is reliable, and almost the same numbers of positive, neutral, and negative tweets are obtained with this combination.

### RQ 2:How effective is our proposed machine learning and deep learning approaches for the classification of sentiment on healthcare tweets?

The dataset contains three classes including positive, negative, and neutral. To train and test models for classification tasks the data is split into 80 to 20 ratios for training and testing, respectively. Experimental results are given in Table 4 where the evaluation is carried out in terms of precision, recall, and F1 score.

**Table 4:** Performance of machine learning models.

Model	Accuracy	Precision	Recall	F1 Score
RF	0.87	0.87	0.78	0.81
LR	0.85	0.87	0.73	0.77
GNB	0.48	0.65	0.58	0.48
ETC	0.88	0.89	0.78	0.82
DT	0.83	0.77	0.75	0.76
KNN	0.60	0.47	0.70	0.47
SGDC	0.86	0.88	0.74	0.78
SVM	0.86	0.86	0.73	0.77
ADA	0.77	0.82	0.67	0.70

 Results indicate that the ETC shows the best performance for sentiment classification with a 0.88 accuracy score which is the highest among all the models. It is followed by the RF which has an accuracy score of 0.87. SVM and SGDC have 0.86 accuracy scores. Results suggest that tree-based classifiers perform better than linear classifiers.

In addition to machine learning models, this study also deployed deep learning models for comparison with machine learning models such as long short term memory (LSTM) and convolutional neural networks (CNN). Both models are selected regarding their reported performance for the task at hand and deployed with state-of-the-art architecture. Each model takes the input through an embedding layer with a 5,000 vocabulary size and 100 output dimensions. In the LSTM model, the embedding layer is followed by a dropout layer with a 0.2 dropout rate and an LSTM with 100 units. The LSTM layer is followed by the dense layer with three neurons and a Softmax activation function. While in CNN, the embedding layer is followed by the 1D CNN layer with 128 filters and 4 ×4 kernel size with rectified linear unit (ReLU) activation function. The CNN layer is followed by the max-pooling layer with a 4 × 4 pool size to extract important features. After the max-pooling layer, we used flatten layer to convert the 3 dimension data to 1 dimension which is followed by the dense layer with three neurons and the Softmax activation function. Both models are compiled with the ‘Adam’ optimizer and categorical_crossentropy loss function. We fitted the model with 100 epochs and 16 batch sizes. The architecture of the used models is shown in Table 5.

**Table 5:** Architecture of deep learning models.

LSTM	CNN
Embedding(5,000,100)	Embedding(5,000,100)
Dropout(0.2)	Dropout(0.2)
LSTM(100)	Conv1D(128, 4,activation=‘relu’)
Dropout(0.2)	MaxPooling1D(pool_size=4)
Dense(32)	Flatten()
Dense(3, activation=‘softmax’)	Dense(3, activation=‘softmax’)
Loss=‘categorical_crossentropy’, optimizer= ‘adam’, epochs=100, batch_size=16	

**Figure 6:**
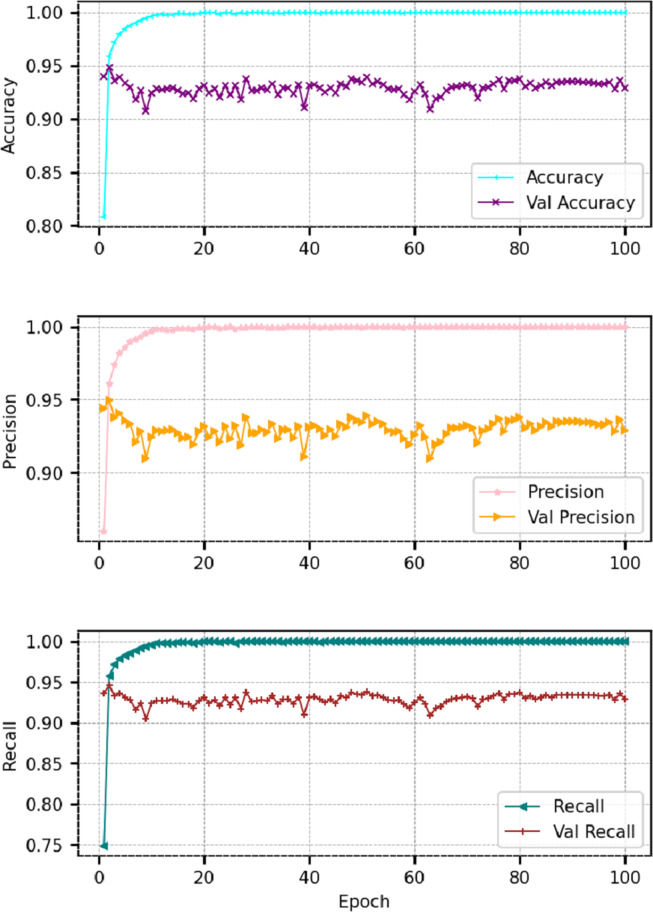
Epoch-wise results for LSTM and CNN models.

Table 6 shows the results for deep learning models and indicates that LSTM performs better as compared to CNN because of its recurrent architecture. Recurrent architecture helps to perform well on sequential text inputs. Figures 6A & 6B show the per epochs results in terms of each evaluation parameter.

**Table 6:** Performance of deep learning models.

Model	Accuracy	Precision	Recall	F1 Score
LSTM	0.93	0.92	0.92	0.96
CNN	0.85	0.87	0.88	0.87
Proposed	0.95	0.96	0.97	0.96

Besides the sentiment analysis using the machine learning and deep learning models, the sentiment frequency distribution indicates that the ratio of positive comments for the health services is substantially higher than the negative sentiments. It shows the higher level of satisfaction of the public regarding the provided services by health institutions.

### Cross-validation to analyze efficacy of proposed model

We employed the K-fold cross-validation methodology in new experiments to verify the results and efficacy of the proposed method. Table 7 demonstrates that ML algorithms, particularly SGD, obtained a mean accuracy of 82% and a standard deviation of ±0.03, demonstrating their efficiency. RF and SVM achieved the same mean accuracy, but SVM obtained a better standard deviation rate. The mean accuracy of DL and CNN models was 84% and 91%, respectively, with ±0.05 and ±0.04 standard deviations. The ML or DL models are not performed excellently using a cross-validation dataset. Cross-validation results demonstrate that the performance of our proposed method is superior, as it attained a remarkable 93% accuracy with a standard deviation of ±0.02.

**Table 7:** Cross-validation results.

**Model**	**Mean Accuracy**	**Standard Deviation (SD)**
RF	0.81	+/- 0.05
LR	0.80	+/- 0.04
GNB	0.55	+/- 0.03
ETC	0.81	+/- 0.06
DT	0.67	+/- 0.00
KNN	0.61	+/- 0.04
SGD	0.82	+/- 0.03
SVM	0.81	+/- 0.02
ADA	0.73	+/- 0.04
CNN	0.84	+/- 0.05
LSTM	0.91	+/- 0.04
Proposed	0.93	+/- 0.02

We performed more experiment analysis using correct and wrong predictions made by the models and validated the efficacy of the proposed model. Table 8 shows the result analysis through correct and wrong predictions. The GNB model attained the highest number of wrong predictions (53%), followed by KNN with 39%. These two models (KNN and GNB) are inefficient and do not contribute to good predictions. Result analysis demonstrates that ML models are incapable of making correct predictions, while DL-based CNN and LSTM models are better than ML in terms of making wrong predictions. CNN attained 15% of its predictions wrong. The proposed LSTM-ETC model attained minimum wrong and maximum correct predictions (5% and 95%). This analysis validates that the proposed model is better to understand and predict sentiments.

**Table 8:** Result regarding correct and wrong predictions on updated dataset.

**Model**	**Correct predictions**	**Wrong predictions**	**Model**	**Correct predictions**	**Wrong predictions**
RF	3,940 (87%)	579 (13%)	SGD	3,925 (87%)	594 (875)
LR	3,860 (85%)	659 (15%)	SVM	3,943 (87%)	576 (13%)
GNB	2,130 (47%)	2,389 (53%)	ADA	3,473 (77%)	1,046 (23%)
ETC	3,978 (88%)	541 (12%)	CNN	3,841 (85%)	678 (15%)
DT	3,755 (83%)	764 (17%)	LSTM	4,222 (93%)	297 (7%)
KNN	2,739 (61%)	1,780 (39%)	Proposed	4,301 (95%)	218 (5%)

**Figure 7:**
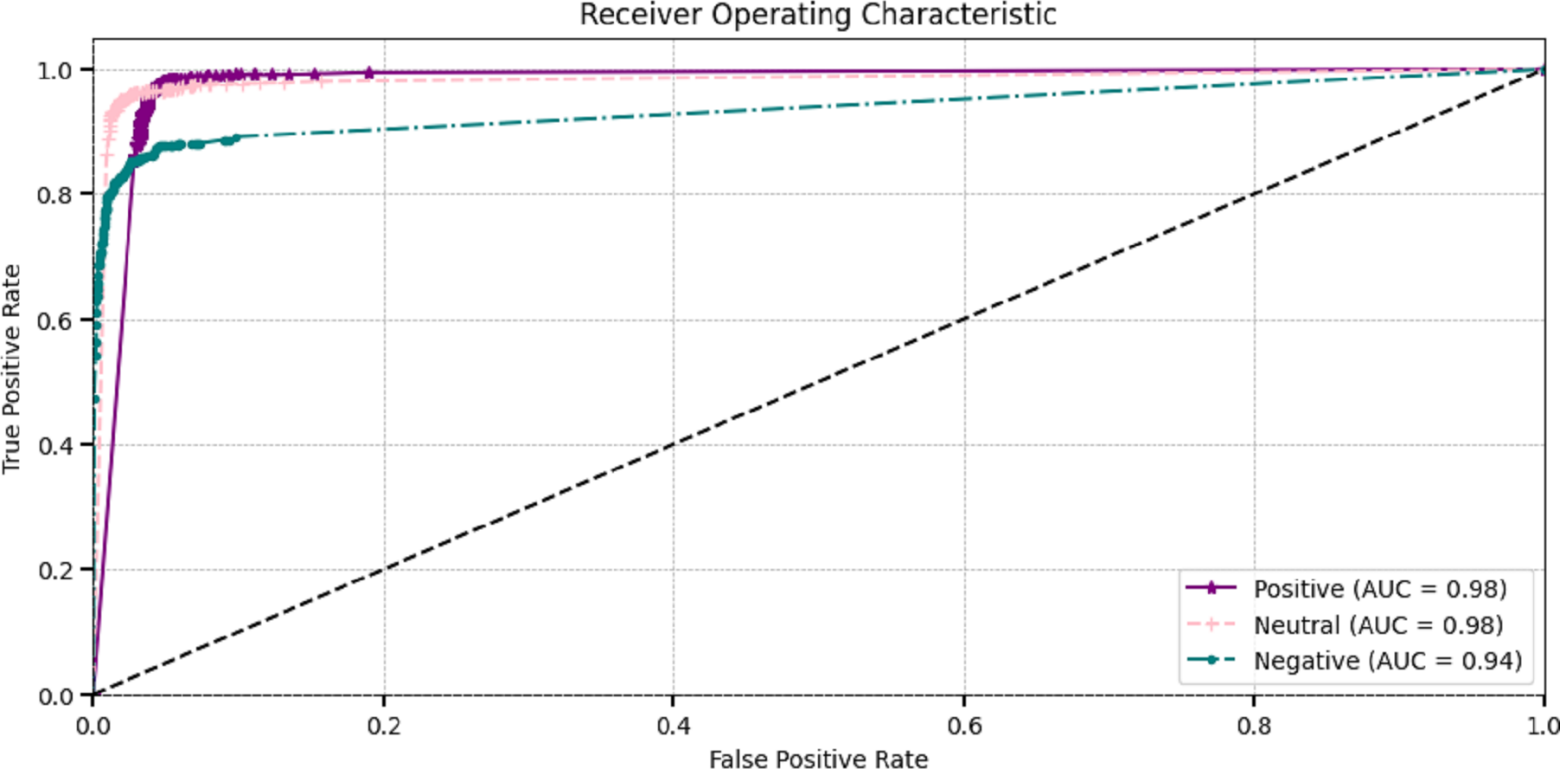
Receiver operating characteristic of proposed method.

The effectiveness of DL models is assessed through ROC curves at different classification factors. The true positive rate and the false positive rate curves demonstrate the effectiveness of ROC-AUC for making the difference between positive and negative classes. The most popular area under the ROC curve (AUC-ROC) is employed to assess the model’s overall performance. They visualize classes very clearly. Figure 7 visualizes the AUC curves of the proposed model, which showed that positive and neutral classes attained superior 0.98 AUC and negative classes attained 0.93.

**Figure 8:**
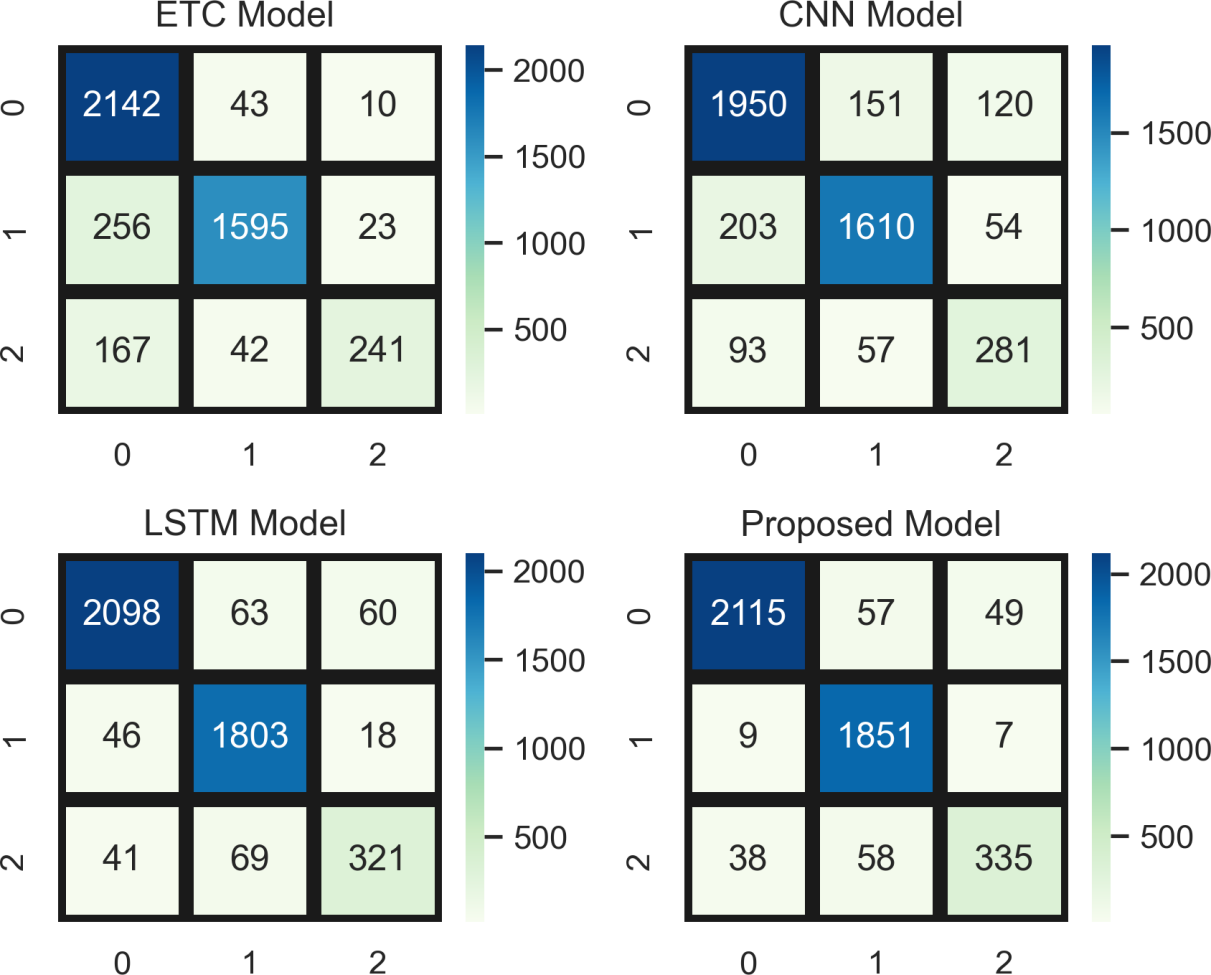
Comparative analysis of top-performing models using the confusion matrix.

Figure 8 indicates a comparative analysis of top-performing models (ETC, CNN, LSTM, and a proposed hybrid model) using the confusion matrix. In the confusion matrix, 0 represents the positive class, 1 represents the neutral class, and 2 represents the neutral class. ETC achieved 2,142 true positives, CNN achieved 1,950, LSTM achieved 2,098, and the proposed model achieved 2,115 true positive values (TPV) for positive class. In addition, the confusion matrix analysis also showed better overall performance than the other three top-performing models with the fewest mistakes.

### Implications of research

**Figure 9:**
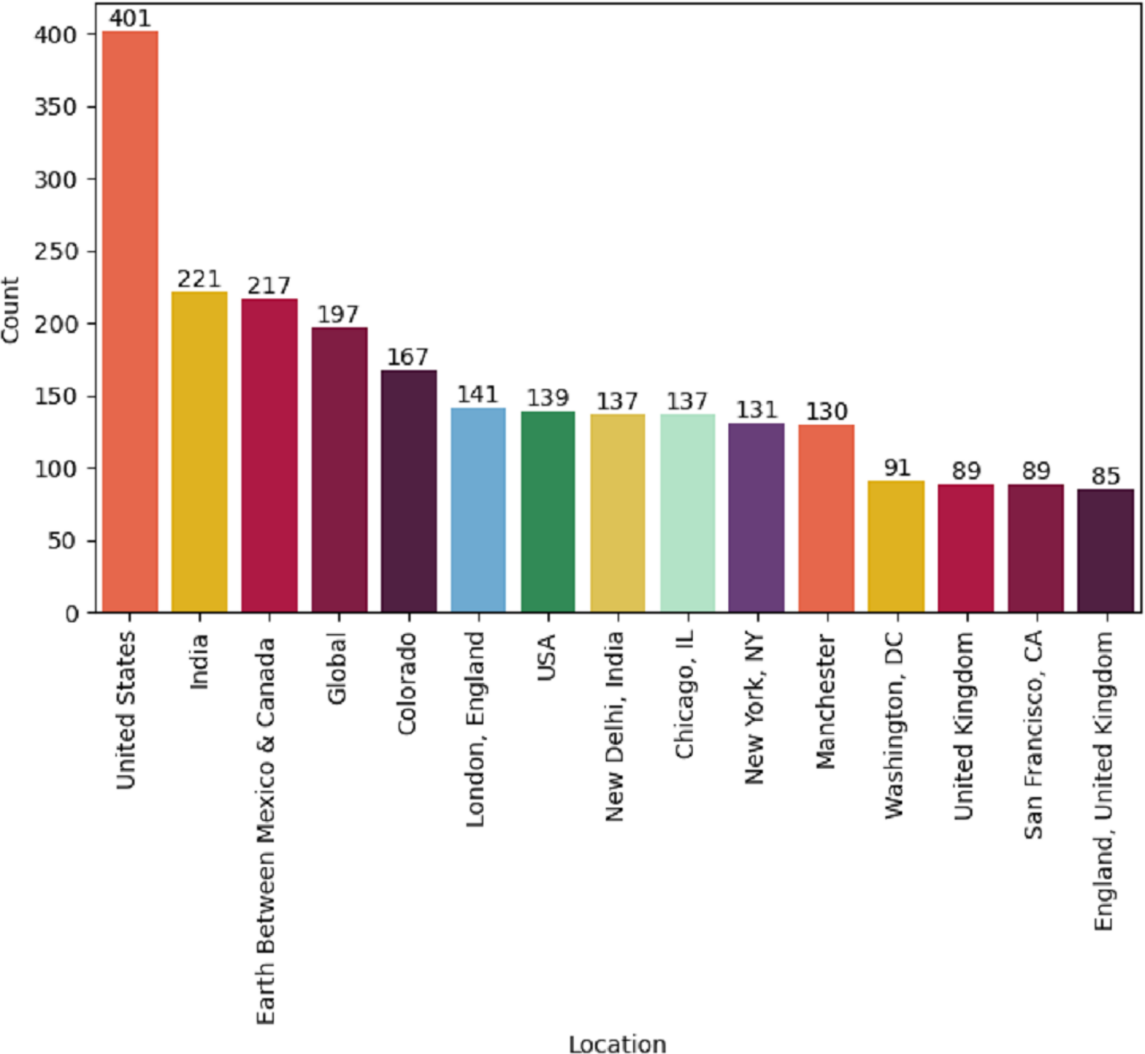
Location-wise tweets posted by worldwide users.

This study can be beneficial to the major stakeholders, including the government, health departments, organizations, policymakers, and patients. They can make policies and enhance or update their services and technological resources by analyzing the satisfaction level of these patients in this study. Also, stakeholders can capture changes and trends in healthcare through the study results. The collected dataset contains tweets worldwide and it’s a limitation of this study because our extracted tweets are from well-developed areas where facilities are good as shown in Fig. 9. There is a lack of tweets from under-developing countries such as India, Pakistan, Afghanistan, Bangladesh, etc. where healthcare facilities are not good. May the inclusion of tweets from these developing countries increase the ratio of negative sentiments.

 Similarly, for the classification approach, the dataset is too small and imbalanced as the ratio of sentiments positive, negative, and neutral is not equal to train the learning models which can affect the validity of machine learning and deep learning models.

**Figure 10:**
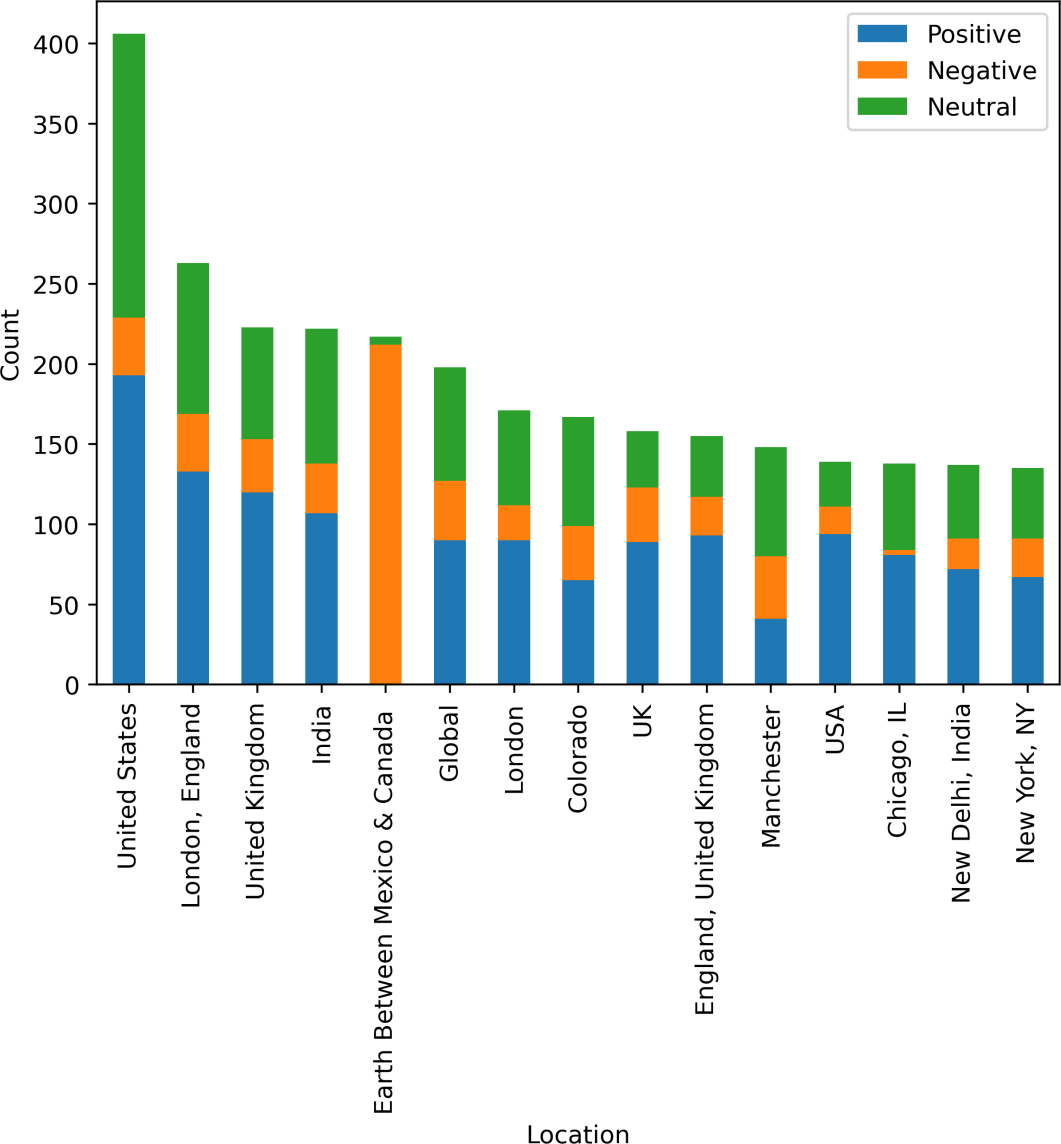
Country-wise analysis of tweets posted by users.

Figure 10 shows country-wise analysis of tweets as positive, negative, and neutral sentiments. In the United States, users have 48% positive opinions about medical services and 0.08% negative opinions about the services. England is on the second number, regarding the tweets posted by the user, and provides 60% positive sentiments and 16% negative sentiments. Between Mexico and Canada, there are limited health facilities that are 0% positive and 97% people dissatisfied with the health services and medical facilities. Figure 10 visualize country wise sentiments of peoples regarding the health services and facilities.

### Performance comparison of proposed model with existing studies

Table 9 shows a comparison between the performance of the proposed model and that of other studies presented in the literature. The authors of the study (Ji, Chun & Geller, 2013) used Facebook tweets and Python NLTK tools to label the tweets. They use different ML models to figure out how people feel, and 87% of the time, they are right. Yang, Lee & Kuo (2016) used MedHelp to get text data for a study, and 13,535 tweets were used as a source. The authors used the AFINN sentiment analyzer and did topic modeling with latent Dirichlet allocation. The authors don’t do anything at all. Gopalakrishnan & Ramaswamy (2017) used a collection of drug reviews that only had about 3,600 reviews. They were 79% accurate in how they put the reviews into classification. The next two studies (Alayba et al., 2017; Rahim et al., 2021) used hand-annotated datasets to classify health tweets and got less accurate results. SuryaPrabha & Balakrishnan (2021), labeled the data with the sentiword-net dictionary, but they also got less accurate results. The suggested methodology produced the best results, utilizing the TextBlob approach on 18,000 custom tweets, Additionally, the proposed model was evaluated and trained using different ML models with Vader lexicons.

**Table 9:** Performance comparison of the proposed model with existing studies.

**Authors**	**Source**	**Dataset**	**Sentiment analyzer**	**Technique**	**Result**
Ji, Chun & Geller (2013)	Facebook	3152	Python nltk	NB, SVM, LR	87%
Yang, Lee & Kuo (2016)	MedHelp	13,535	AFINN	LDA	–
Gopalakrishnan & Ramaswamy (2017)	Drug review	3,600	N/A	SVM	79.8%
Alayba et al. (2017)	Twitter	2,026	Manual annotation	NB, SVM, LR	85.2%
Rahim et al. (2021)	Facebook	1,793	Manual annotation	NB, SVM, LR	90%
SuryaPrabha & Balakrishnan (2021)	MedHelp	1,980	Sent-wordnet	SVM	82%
Saad et al. (2021)	NHS	50,716	AFINN and NHSdict	SVM,DT,KNN,	63%
This study	Twitter	18,000	TextBlob	LSTM+ETC	95%

### Discussion and limitations

In this research, we determine how to analyze patients’ satisfaction levels with medical services and sentiment using Twitter data. Sentiment analysis can be used to identify large data sets of free-text comments related to healthcare improvement. Twitter has established itself as an important element of the social media landscape, where people interact and share their views and concerns regarding public, governmental, and social services. These views are replete with ample information to analyze public opinion, formulate policies, comprehend the effect of events, and devise novel solutions to specific issues. This study provides an early attempt to monitor public attitudes toward healthcare services through the use of novel data sources as well as a technique for leveraging social media data. This study leverages many well-known machine learning and deep learning models to analyze sentiments regarding medical services. A comprehensive discussion is presented of the performances and behaviors of the conventional and deep learning approaches used.

Experiments are performed using several well-known machine learning models, including support vector machines, logistic regression, Gaussian naive Bayes, extra tree classifiers, k-nearest neighbor, random forests, decision trees, and AdaBoost. Results suggest that ETC achieves the best performance among machine learning models, with an accuracy score of 0.88. However, the proposed model surpasses it with an accuracy of 0.95 when predicting positive, negative, and neutral sentiments. The proposed model shows superior performance. The majority of people are satisfied with the services, and a very small number of people are dissatisfied with them.

Twitter can offer significant benefits to organizations and healthcare departments by serving as a method for evaluating healthcare services. In this era, healthcare departments and several other organizations possess the capability to actively monitor social media platforms such as Twitter. The organization faces obstacles in its ability to effectively monitor and analyze lengthy tweets or a large volume of tweets to improve decision-making and get insights into prevalent health-related issues. The analytical process is time-consuming, and the findings obtained are inaccurate in terms of the manual examination of tweets. The patients provide feedback about the health facilities and resources, as well as using online platforms to schedule appointments, among other activities. This study examines the levels of patient satisfaction with medical services. The utilization of Twitter data to extract the thoughts and attitudes conveyed by its users. Organizations have the potential to get valuable insights by analyzing tweets or doing sentiment analysis on articles about various health-related topics. The tweets provided data on the patients, who were routinely queried about their encounters with the medical personnel, hospital services, and other healthcare-related establishments. The analysis of Twitter’s outcomes can provide valuable insights for healthcare organizations seeking to improve the quality of their services. The prevailing allocation of resources is presently concentrated on improving the well-being of medical practitioners through the pursuit of machine learning research and development utilizing extensive quantities of health-related data. The organizations can make significant decisions, and hospital staff communicate with their patients more politely and provide correct information.

There are some limitations to this study, including the fact that the dataset was collected from different parts of the globe, the difficulty of analyzing every country due to their unique healthcare systems, and the potential bias of generalizing the results. Second, the collected healthcare services and facilities dataset is small for training transformer-based deep learning models without overfitting.

## Conclusion

Twitter has established itself as an important element of the social media landscape, where people interact and share their views and concerns regarding public, governmental, and social services. Such views are replete with ample information to analyze public opinion, formulate policies, comprehend the effect of events, and devise novel solutions to specific issues. This article leverages many well-known machine learning and deep learning models to analyze sentiments regarding medical services. Results suggest that ETC achieves the best performance among machine learning models with a 0.88 accuracy score, however, the proposed model surpasses it with a 0.95 accuracy when predicting the positive, negative, and neutral sentiments. The proposed model makes only 5% wrong predictions compared to GNB. The proposed model attained a superior AUC of 0.98 for positive and neutral tweets.

In future work, we will work on two problems: one we will perform sentiment analysis by dividing the dataset into two categories which are developing counties and developed counties. We will collect the tweets dataset according to location so a fair analysis can be performed. Second, we will collect a large and balanced dataset for the training of models so that overfitting chances can be reduced.

## Supplemental Information

10.7717/peerj-cs.1697/supp-1 Supplemental Information 1 Implementation code and data

## Additional Information and Declarations

### Data Availability

The following information was supplied regarding data availability: The data and implementation code is available in the Supplemental File.
